# Daily Physical Activity, Sports Participation, and Executive Function in Children

**DOI:** 10.1001/jamanetworkopen.2024.49879

**Published:** 2024-12-17

**Authors:** Lu Yang, Eva Corpeleijn, Esther Hartman

**Affiliations:** 1 Department of Human Movement Sciences, University Medical Center Groningen, University of Groningen, Groningen, the Netherlands; 2 Department of Epidemiology, University Medical Center Groningen, University of Groningen, Groningen, the Netherlands

## Abstract

**Importance:**

It is unclear whether daily physical activity and sports participation relate to executive function (EF) in children.

**Objective:**

To explore associations between early-life physical activity, sports participation, and executive function in primary school children.

**Design, Setting, and Participants:**

Data for this cohort study were collected from April 2006 to December 2017 from the Groningen Expert Center for Kids with Obesity (GECKO) Drenthe birth cohort, which includes Northern Dutch children. Preschool children aged 5 to 6 years and primary school children aged 10 to 11 years with valid EF, daily physical activity, and sports participation data were included in the analysis. Data were analyzed from May 2023 to February 2024.

**Exposures:**

At ages 5 to 6 years, daily physical activity was measured with accelerometry. At ages 10 to 11 years, sports participation data were collected using the Short Questionnaire to Assess Health–Enhancing Physical Activity.

**Main Outcomes and Measures:**

Executive function was assessed using the Behavior Rating Inventory of Executive Function at ages 10 to 11 years.

**Results:**

A total of 880 children (470 female [53.4%]; mean [SD] age at EF measurement, 11.1 [0.4] years) were included. Moderate to vigorous physical activity at ages 5 to 6 years did not correlate with EF at ages 10 to 11 years (eg, Global Executive Composite [GEC]: β, 0.16; 95% CI, −0.21 to 0.53). Higher levels of light physical activity at ages 5 to 6 years were associated with poorer inhibitory control (β, 0.86; 95% CI, 0.24-1.47) and self-monitoring (β, 0.79; 95% CI, 0.09-1.48) at ages 10 to 11 years, while greater sedentary time was associated with better inhibitory control (β, −0.62; 95% CI, −1.08 to −0.16) and self-monitoring (β, −0.57; 95% CI, −1.09 to −0.06). Compared with children engaged in individual sports, team sports participants exhibited better overall EF (mean difference [SE], −3.03 [0.81]), behavior regulation (mean difference [SE], −3.39 [0.77]), and metacognition (mean difference [SE], −2.55 [0.81]). Children involved in both types of sports vs only individual sports had better EF (GEC: mean difference [SE], −2.66 [0.93]).

**Conclusions and Relevance:**

In this cohort study of Dutch children, early-life moderate to vigorous physical activity was not associated with EF in middle childhood. Children participating in team sports at ages 10 to 11 years consistently exhibited superior EF compared with participants in individual sports.

## Key Points

**Question:**

Are daily physical activity in early life and sports participation later in childhood associated with executive function in middle childhood?

**Findings:**

In this cohort study of 880 children, moderate to vigorous physical activity at ages 5 to 6 years was not associated with executive function at ages 10 to 11 years. Compared with participants in individual sports, children involved in team sports or both types of sports exhibited superior executive function at ages 10 to 11 years.

**Meaning:**

The findings suggest that to enhance children’s executive function through sports, it is important to consider the type of sports.

## Teaser

This cohort study investigates associations of physical activity at ages 5 to 6 years and sports participation later in childhood with executive function at ages 10 to 11 years.

## Introduction

Physical activity is well known to benefit physical and mental well-being and to contribute to healthy growth and development in children.^1^ It serves as a promising nonpharmacologic approach to enhancing cognitive function.^2^ Executive function (EF), the high-order cognitive processes necessary for purposeful, problem-solving, goal-directed behavior, has been shown to improve in association with physical exercise interventions across the lifespan.^3^

Evidence on benefits of physical activity for EF primarily derives from intervention studies. Short-term physical activity was associated with improved inhibition but not working memory or flexibility in children aged 6 to 12 years,^4^ while it was also reported to be associated with benefits in inhibition, working memory, and flexibility in individuals aged 5 to 18 years.^5^ Long-term physical activity was associated with enhanced working memory and flexibility but not inhibition in a meta-analysis among children aged 6 to 12 years,^4^ and another meta-analysis indicated that chronic physical activity was associated with improved overall EF, including inhibition, in children aged 6 to 17 years.^6^ Overall, these studies suggest that physical activity interventions are associated with improved EF, although evidence on specific EF subdomains may vary.

However, findings from interventional settings may not directly translate to daily physical activity. Research on the impact of daily physical activity on EF in children is limited. Luo et al^7^ found that preschool children with higher moderate to vigorous physical activity (MVPA) levels had better inhibition. In addition, Zeng et al^8^ reported that less sedentary time and higher physical activity levels correlated with better EF in children aged 6 to 12 years. These cross-sectional studies suggested correlations between physical activity and EF, but stronger evidence is needed. Executive function emerges in early life and accelerates during school years. It is worth exploring whether early-life physical activity influences EF during this formative period. Furthermore, while long-term associations of physical activity with EF have been studied in adolescents and adults,^9,10^ evidence for young children remains limited. Thus, this study aimed to examine whether daily physical activity in early life is associated with EF in later childhood.

Daily physical activity originates from various sources, with sports being a significant contributor. A study of German children aged 6 to 10 years found that they achieved 28% to 31% MVPA during organized sports and were more likely to meet the World Health Organization recommendation of 60 daily minutes of MVPA by participating in sports at least twice a week.^11^ Additionally, sports that develop motor skills may enhance EF, as motor skills mediate the association between physical activity and EF.^12^ Therefore, exploring the associations between sports participation and EF in childhood is valuable. In Europe, 52% to 73% of children aged 11 years were in sports clubs.^13^ In the Netherlands, 87% of children aged 6 to 11 years participated in sports regularly in 2022, and 69% had sports club memberships—rates higher than in other age groups.^14^ Therefore, the current study focused on sports in Dutch children aged 10 to 11 years.

Executive function improvement through sports may depend on characteristics inherent to activities. Sports with cognitive demands are beneficial for EF. Previous studies indicated that different sports may impact EF distinctly. For example, tennis players exhibited better inhibition than swimmers.^15^ However, evidence for a relationship between different sports and EF in childhood is scarce. A meta-analysis found positive associations between sports interventions and EF,^16^ but it was limited to small studies. Additionally, previous studies primarily focused on a narrow range of sports. De Waelle et al^17^ observed that young females participating in basketball, volleyball, soccer, korfball, or hockey showed superior EF than those in self-paced sports. However, children typically have access to a variety of sports and even participate in multiple sports simultaneously.

In response to those knowledge gaps, the current study aimed to examine the associations between daily physical activity, sports participation, and EF in children. Specifically, the study sought to assess whether daily physical activity in early life is associated with later EF and to examine whether types, durations, and number of sports played during middle childhood are associated with EF, covering a broad array of sports in everyday settings.

## Methods

### Study Design

The Groningen Expert Center for Kids with Obesity (GECKO) Drenthe birth cohort includes Northern Dutch children. Data for the current cohort study were collected from April 2006 to December 2017. Further information is available elsewhere.^18^ Daily physical activity was measured in preschool children aged 5 to 6 years. Executive function and sports participation were evaluated in primary school children aged 10 to 11 years (eFigure in Supplement 1). This study was approved by the Medical Ethics Committee of University Medical Center Groningen and followed the Declaration of Helsinki guidelines.^19^ Written informed consent for participation was given by parents or guardians. This study followed the Strengthening the Reporting of Observational Studies in Epidemiology (STROBE) reporting guideline.

### Daily Physical Activity

Participants aged 5 to 6 years wore an ActiGraph GT3X accelerometer on the right hip during waking hours for 4 consecutive days except during water-based activities. Data were collected using 30-Hz frequency and analyzed with a 15-second epoch. Physical activity intensities were computed using cutoff points: sedentary time (≤819 counts per minute [cpm]), light physical activity (LPA; 820-3907 cpm), moderate physical activity (MPA; 3908-6111 cpm), vigorous physical activity (≥6112 cpm), and MVPA (≥3908 cpm).^20^ A valid measurement required wearing time of at least 600-min/d on at least 3 days.

### Sports Participation

The Short Questionnaire to Assess Health—Enhancing Physical Activity (SQUASH) was used to assess sports participation among children aged 10 to 11 years. It has been validated and widely used.^21,22^ Parents reported up to 4 types of sports, number of days of participation per week, and average time per day for each sport. We classified sports into team and individual sports.^23^ Team sports were defined as activities requiring group collaboration and impossible for a child to perform alone. Individual sports referred to activities allowing independent participation.

### Executive Function

The Behavior Rating Inventory of Executive Function (BRIEF), Dutch translation was used to assess EF at ages 10 to 11 years.^24^ This parental questionnaire yields 8 subscales: (1) inhibition (controls impulses), (2) shift (adapts to changes), (3) emotional control (regulates emotional responses), (4) initiate (starts tasks without prompting), (5) working memory (holds and manipulates information), (6) plan and organize (develops strategies and organizes tasks), (7) organization of materials (keeps belongings well organized), and (8) monitor (tracks and self-evaluates performance). It generates Behavioral Regulation Index (BRI; self-regulation abilities), Metacognition Index (MI; problem-solving capacity), and Global Executive Composite (GEC; overall EF) subscale scores (eTable 1 in Supplement 1). All scores were converted into T scores based on sex and age.^24^ Higher T scores indicate greater difficulties in EF, with scores of 65 or above reaching a clinical threshold that may require intervention. The internal consistency of BRIEF was confirmed, with a Cronbach α ranging from 0.83 to 0.97 (eTable 2 in Supplement 1). Inconsistency and negativity for study cases were calculated to identify response biases.

### Other Factors

Sex and age were reported by questionnaires. Height and weight were measured by nurses.^25^ Body mass index (BMI) was calculated accordingly. Season of physical activity and sports was determined by dates. Parental questionnaires collected data on outdoor play time, computer time, TV watching time, and socioeconomic status, including dwelling type, household income, and family size. Maternal and paternal educational levels were reported during pregnancy and included (1) no education or lower general secondary education; (2) senior secondary vocational, higher general secondary education, or preuniversity education; and (3) higher vocational or university-level education.

### Statistical Analysis

Sample characteristics were described as frequency and percentage for categorical variables, means with SDs for normally distributed variables, and medians with IQRs for nonnormally distributed variables. Spearman rank correlations were calculated. Multiple linear regression models were fitted for physical activity and adjusted for age, sex, outdoor playtime, number of children in the family, maternal educational level, accelerometer wear time, and season. Furthermore, children were grouped first by the number and then by the type of sports. Analysis of variance was used to compare EF among groups. Next, multiple linear regression models were applied for sport types and adjusted for age, BMI, total time spent participating in sports, outdoor play time, computer time, maternal educational level, and number of children in the family. Missing values for BMI and number of children in the family were replaced with group means. The same models were used for specific sports. A sensitivity analysis was performed by repeating multiple linear analyses on a restricted sample excluding sports showing contrasting correlations with EF. Statistical significance was set at 2-sided *P* < . 05. A Bonferroni correction threshold of *P* < .008 was used for models with sport types. Analyses were performed from May 2023 to February 2024 using SPSS, version 28.0 (IBM Corp).

## Results

### Study Population

This study included 880 children (470 females [53.4%]; 410 males [46.6%]; mean [SD] age at EF measurement, 11.1 [0.4] years) (Figure). At ages 5 to 6 years, children spent most of their day in sedentary behaviors, with a mean (SD) LPA time of 263.4 (38.5) min/d and median MVPA time of 61.2 min/d (IQR, 46.6-80.0 min/d) (Table 1). At ages 10 to 11 years, children spent a mean (SD) of 4.6 (3.7) h/wk participating in sports, with 771 (87.6%) participating in at least 1 sport. In total, there were 11 types of team sports and 22 types of individual sports (eTable 3 in Supplement 1).

**Figure.**
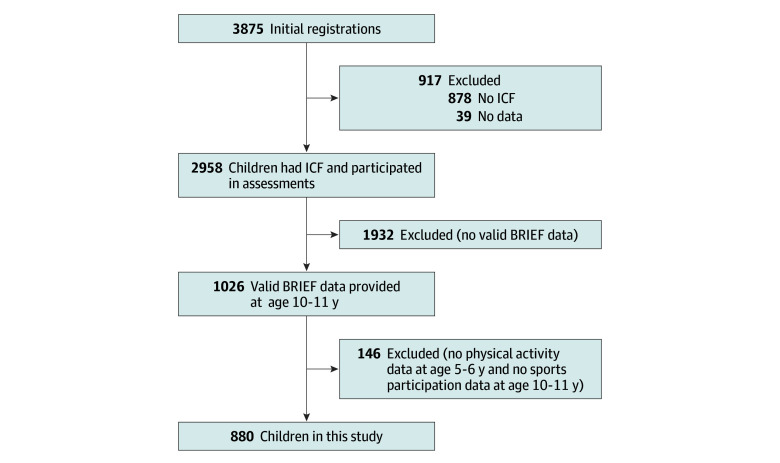
Flowchart of Participant Enrollment BRIEF indicates Behavior Rating Inventory of Executive Function; ICF, informed consent form.

**Table 1.** Descriptive Characteristics of the Study Population

Characteristic	Children (N = 880)^a^
Sex	
Female	470 (53.4)
Male	410 (46.6)
Age at PA assessment, mean (SD), y	5.8 (0.3)
Age at EF measurement, mean (SD), y	11.1 (0.4)
BMI, mean (SD)	17.5 (2.4)
Computer time at ages 10-11 y, median (IQR), h/d	2.3 (1.7-3.0)
Outdoor play time at ages 10-11 y, median (IQR), min/d	77.1 (45.0-115.7)
No. of children in household	
1	335 (38.1)
2	331 (37.6)
>2	154 (17.5)
Maternal educational level^a^	
Low	349 (39.7)
Moderate	367 (41.7)
High	105 (11.9)
Daily PA at ages 5-6 y, min/d	
Overall, median (IQR)	702.6 (33.7)
Sedentery, median (IQR)	373.4 (336.3-411.9)
Light, mean (SD)	263.4 (38.5)
Medium, median (IQR)	43.5 (33.7-55.1)
Vigorous, median (IQR)	16.6 (11.6-24.2)
Medium to vigorous, median (IQR)	61.2 (46.6-80.0)
Time spent in sports at ages 10-11 y, mean (SD), h/wk	4.6 (3.7)
No. of sports played (n = 823)	
0	52 (6.3)
1	441 (53.6)
2	230 (27.9)
3	84 (10.2)
4	16 (1.9)
Type of sport (n = 823)	
No sport	52 (6.3)
Individual sport	312 (37.9)
Team sport	270 (32.8)
Both individual and team sports	189 (23.0)
Specific sports (n = 823)	
Soccer	287 (34.9)
Gymnastics	178 (21.6)
Dancing	92 (11.2)
Martial arts	73 (8.9)
Horse riding	71 (8.6)
Swimming	71 (8.6)
Volleyball	58 (7.0)
Tennis	44 (5.3)
Hockey	44 (5.3)
Other sports, range	1 (0.1) to 30 (3.6)

Abbreviations: BMI, body mass index (calculated as weight in kilograms divided by height in meters squared); EF, executive function; PA, physical activity.

^a^ Data are presented as number (percentage) of children unless otherwise indicated.

^b^ Low was defined as no education or lower general secondary education; moderate as senior secondary vocational education, higher general secondary education, or preuniversity education; and high as higher vocational education or university-level education.

### Daily Physical Activity and EF

No correlation was observed between MVPA and BRIEF T scores, but a correlation was found for MPA (eTable 4 in Supplement 1). In line with this, MVPA was not associated with EF (eg, GEC: β, 0.16; 95% CI, −0.21 to 0.53), but a higher level of MPA at ages 5 to 6 years was associated with worse inhibition (β, 0.66; 95% CI, 0.12-1.20) and monitor (β, 0.69; 95% CI, 0.08-1.30) at ages 10 to 11 years. Similarly, more LPA at 5 to 6 years was associated with poorer inhibition (β, 0.86; 95% CI, 0.24-1.47) and monitor (β, 0.79; 95% CI, 0.09-1.48) at 10 to 11 years. However, greater sedentary time at 5 to 6 years was associated with better inhibition (β, −0.62; 95% CI, −1.08 to −0.16) and monitor (β, −0.57; 95% CI, −1.09 to −0.06) at 10 to 11 years (Table 2).

**Table 2.** Associations Between Daily Physical Activity at Age 5 to 6 Years and Executive Function at Age 10 to 11 Years

BRIEF scale	β (95% CI)^a^				
	SED	LPA	MVPA	MPA	VPA
Index					
GEC	−0.36 (−0.88 to 0.15)	0.48 (−0.22 to 1.17)	0.16 (−0.21 to 0.53)	0.55 (−0.05 to 1.16)	−0.16 (−0.87 to 0.55)
BRI	0.25 (−0.86 to 0.12)	0.34 (−0.15 to 1.19)	0.18 (−0.22 to 0.48)	0.30 (0.00 to 1.16)	0.35 (−0.98 to 0.37)
MI	−0.36 (−0.88 to 0.15)	0.46 (−0.24 to 1.15)	0.17 (−0.20 to 0.54)	0.49 (−0.11 to 1.10)	−0.04 (−0.75 to 0.67)
Subscales					
Inhibition	−0.62 (−1.08 to −0.16)	0.86 (0.24 to 1.47)	0.23 (−0.10 to 0.56)	0.66 (0.12 to 1.20)	−0.04 (−0.68 to 0.59)
Shift	0.11 (−0.38 to 0.60)	−0.09 (−0.76 to 0.57)	−0.08 (−0.43 to 0.27)	0.17 (−0.41 to 0.75)	−0.53 (−1.20 to 0.14)
Emotional control	−0.29 (−0.76 to 0.18)	0.38 (−0.25 to 1.02)	0.12 (−0.21 to 0.46)	0.50 (−0.06 to 1.05)	−0.22 (−0.87 to 0.42)
Initiate	−0.21 (−0.72 to 0.30)	0.29 (−0.40 to 0.98)	0.08 (−0.28 to 0.44)	0.13 (−0.47 to 0.74)	0.11 (−0.59 to 0.81)
Working memory	−0.33 (−0.83 to 0.17)	0.36 (−0.31 to 1.03)	0.20 (−0.15 to 0.56)	0.51 (−0.07 to 1.10)	0.06 (−0.62 to 0.75)
Plan and organize	−0.32 (−0.83 to 0.19)	0.36 (−0.34 to 1.06)	0.19 (−0.18 to 0.56)	0.52 (−0.09 to 1.13)	0.00 (−0.71 to 0.71)
Organization of materials	−0.21 (−0.70 to 0.28)	0.32 (−0.34 to 0.98)	0.05 (−0.30 to 0.40)	0.22 (−0.35 to 0.80)	−0.11 (−0.78 to 0.56)
Monitor	−0.57 (−1.09 to −0.06)	0.79 (0.09 to 1.48)	0.22 (−0.15 to 0.59)	0.69 (0.08 to 1.30)	−0.11 (−0.82 to 0.60)

Abbreviations: BRI, Behavioral Regulation Index; BRIEF, Behavior Rating Inventory of Executive Function; GEC, Global Executive Composite; LPA, light physical activity; MI, Metacognition Index; MPA, moderate physical activity; MVPA, moderate to vigorous physical activity; SED, sedentary behavior; VPA, vigorous physical activity.

^a^ Adjusted for exact age, body mass index, outdoor play time, computer time, maternal educational level, number of children in household, accelerometer wear time, and season. Fully adjusted models are presented here, and unadjusted models are included in eTable 6 in Supplement 1.

### Number of Sports, Time Spent in Sports, and EF

The number of sports in which children participated had no correlation with EF (eTable 4 in Supplement 1), and EF did not differ among groups categorized by number of sports (eTable 5 in Supplement 1). Conversely, more total time spent in sports was associated with better behavior regulation, specifically shift and emotional control as well as working memory (eTable 4 in Supplement 1).

### Types of Sports and EF

Some sport types were correlated with EF (eTable 4 in Supplement 1). Compared with children only engaged in individual sports, those exclusively engaged in team sports had lower scores in GEC (mean difference [SE], −3.03 [0.81]; *P* < .001), BRI (mean difference [SE], −3.39 [0.77]; *P* < .001), and MI (mean difference [SE], −2.55 [0.81]; *P* = .002) as well as lower scores in subscales indicating better EF (Table 3). In the sensitivity analysis, after excluding sports with contrasting correlations with EF (eTable 4 in Supplement 1), the differences in GEC and BRI scores between team and individual sports remained consistent (eTable 8 in Supplement 1). Similarly, participants in both team and individual sports showed better overall EF (mean score difference [SE], −2.66 [0.93]) and behavioral regulation (mean score difference [SE], −2.73 [0.88]) compared with participants in individual sports only (Table 3). In contrast, no EF differences were observed between children in team sports and those in both types of sports (eTable 9 in Supplement 1). Moreover, children who played sports did not differ in EF compared with those who did not (eTable 10 in Supplement 1).

**Table 3.** Associations Between Type of Sports and Executive Function Among 823 Children^a^

BRIEF scale	No sport vs individual sport		Team sport vs individual sport		Both vs individual sport	
	MD (SE)	*P* value	MD (SE)	*P* value	MD (SE)	*P* value
Index						
GEC	−1.55 (1.48)	.30	−3.03 (0.81)	<.001	−2.66 (0.93)	.004
BRI	−1.19 (1.40)	.40	−3.39 (0.77)	<.001	−2.73 (0.88)	.002
MI	−1.62 (1.48)	.27	−2.55 (0.81)	.002	−2.33 (0.93)	.01
Subscales						
Inhibition	−0.51 (1.30)	.70	−2.29 (0.71)	.001	−1.96 (0.81)	.02
Shift	−0.81 (1.39)	.56	−2.64 (0.76)	.001	−2.05 (0.87)	.02
Emotional control	−1.16 (1.30)	.37	−2.72 (0.71)	<.001	−2.29 (0.82)	.005
Initiate	−1.08 (1.44)	.46	−2.73 (0.79)	.001	−2.18 (0.90)	.02
Working memory	−2.19 (1.40)	.12	−2.05 (0.77)	.008	−1.67 (0.88)	.06
Plan and organize	−1.71 (1.46)	.24	−1.82 (0.80)	.02	−1.49 (0.92)	.10
Organization of materials	−0.15 (1.33)	.91	−1.71 (0.73)	.02	−2.22 (0.84)	.01
Monitor	−1.23 (1.48)	.41	−2.25 (0.81)	.005	−1.78 (0.93)	.06

Abbreviations: BRI, Behavioral Regulation Index; BRIEF, Behavior Rating Inventory of Executive Function; GEC, Global Executive Composite; MD, mean difference; MI, Metacognition Index.

^a^ Analyses were adjusted for exact age, body mass index, outdoor play time, computer time, maternal educational level, number of siblings, and time spent in sports. Fully adjusted models are presented here. Unadjusted models are reported in eTable 7 in Supplement 1.

Furthermore, associations between specific sports and EF varied (Table 4). Playing soccer vs not playing soccer was associated with better overall EF, behavior regulation, and metacognition. Playing volleyball vs not playing volleyball was only associated with better inhibition and monitor scores. Compared with no gymnastics participation, participating in gymnastics was associated with better metacognition. However, compared with no participation in the respective sport, engaging in martial arts was associated with poorer inhibition and swimming was associated with worse inhibition and emotional control.

**Table 4.** Associations Between Specific Sports and Executive Function

BRIEF scale	Team sports^a^				Individual sports^a^					
	Soccer		Volleyball		Gymnastics		Martial arts		Swimming	
	MD (SE)^b^	*P* value	MD (SE)^b^	*P* value	MD (SE)^b^	*P* value	MD (SE)^b^	*P* value	MD (SE)^b^	*P* value
Index										
GEC	−2.34 (0.74)	.002	−1.79 (1.31)	.17	−1.55 (0.83)	.06	2.22 (1.18)	.06	2.39 (1.23)	.053
BRI	−1.90 (0.70)	.007	−2.36 (1.25)	.06	−0.89 (0.79)	.26	2.02 (1.12)	.07	2.68 (1.17)	.02
MI	−2.38 (0.73)	.001	−1.28 (1.31)	.33	−1.73 (0.82)	.04	2.16 (1.17)	.07	1.88 (1.23)	.13
Subscales										
Inhibition	−0.71 (0.65)	.27	−2.85 (1.14)	.01	−0.87 (0.73)	.23	2.04 (1.03)	.048	2.14 (1.08)	.048
Shift	−1.58 (0.69)	.02	−1.64 (1.23)	.18	−0.04 (0.78)	.96	1.36 (1.10)	.22	2.05 (1.16)	.08
Emotional control	−1.48 (0.65)	.02	−1.77 (1.16)	.13	−1.39 (0.73)	.06	1.76 (1.04)	.09	2.19 (1.09)	.04
Initiate	−1.92 (0.72)	.01	0.22 (1.28)	.87	−0.48 (0.81)	.55	1.41 (1.15)	.22	2.11 (1.20)	.08
Working memory	−2.80 (0.69)	<.001	0.81 (1.24)	.51	−0.99 (0.78)	.21	2.00 (1.11)	.07	1.64 (1.17)	.16
Plan and organize	−1.68 (0.73)	.02	−1.53 (1.29)	.24	−1.56 (0.82)	.06	1.86 (1.16)	.11	1.04 (1.21)	.39
Organize materials	−1.86 (0.66)	.005	−2.04 (1.18)	.08	−1.68 (0.74)	.02	1.23 (1.06)	.24	0.53 (1.11)	.63
Monitor	−1.61 (0.74)	.03	−2.93 (1.30)	.03	−2.05 (0.82)	.01	1.34 (1.17)	.25	2.14 (1.23)	.08

Abbreviations: BRI, Behavioral Regulation Index; BRIEF, Behavior Rating Inventory of Executive Function; GEC, Global Executive Composite; MD, mean difference; MI, Metacognition Index.

^a^ Adjusted for exact age, body mass index, outdoor play time, computer time, maternal educational level, number of siblings, and time spent in sports.

^b^ Mean (SE) difference for comparison between children participating in the given sport and those not engaged in that sport.

## Discussion

This study aimed to examine associations between daily physical activity, sports participation, and EF in children. It showed no association between MVPA at ages 5 to 6 years and EF at ages 10 to 11 years. Higher levels of LPA and MPA at ages 5 to 6 years were associated with poorer EF at ages 10 to 11 years, while greater sedentary time at ages 5 to 6 years was associated with better EF at ages 10 to 11 years. For sports, more total time playing sports was significantly associated with better EF, and participants in team sports had better EF than those in individual sports.

Our findings suggest that early-life MVPA may not be associated with EF in middle childhood, which is inconsistent with previous evidence of an association between higher MVPA and better inhibition.^7,26^ However, those cross-sectional studies did not address prospective associations as our study did. In this study, the lack of associations may have stemmed from diverse sources of MVPA. Accelerometers can quantify physical activity but cannot differentiate between types. Research indicates that only cognitively engaging activities, rather than all types of activities, may benefit EF.^27^ Thus, varying effects of different activities on EF might obscure a relationship between MVPA and EF. Furthermore, the quantity and quality of physical activities change from preschool to primary school.^28,29^ Since EF can be impacted by short-term exercise,^5^ early-life MVPA may not be associated with later EF.

We observed that higher LPA and MPA in early childhood were associated with poorer EF. Previous research showed that more MPA in early life was associated with hyperactivity in middle childhood.^30^ In addition, children with attention-deficit/hyperactivity disorder exhibited higher activity levels and weaker EF.^31,32^ This might explain the observed negative associations between MPA and EF. No other studies to our knowledge have reported on associations between LPA and EF. Our results highlight the need to understand the effects of LPA and MPA, as these crucial components of daily physical activity have not been deeply investigated.

Our finding that sedentary behavior was associated with improved inhibition aligns with previous research,^9^ but other studies reported no association or negative associations.^33,34^ This inconsistency may stem from different levels of cognitive activity involved in various sedentary behaviors. For example, reading correlated with higher brain connectivity in cognitive control regions, whereas screen time was associated with lower brain connectivity.^35^ Thus, future research should consider differences in sedentary behavior when examining its impacts.

Our study found that type of sport was associated with EF. To our knowledge, this was the first study to compare whether different sport types are associated with EF in children while also considering combinations of sports. Our findings showed that combining team and individual sports had consistent associations with better EF. This suggests that children with better EF may engage in multiple sports or that combining sports types may enhance EF more than a single sport. These assumptions should be tested in the future. Conversely, this study found no significant difference in EF between children who participated in sports and those who did not. The small number of nonparticipants in sports limited the power to draw firm conclusions, and we lacked information on children’s reasons for not participating in sports. Thus, this result should be interpreted with caution.

The associations between team sports and EF are supported by a longitudinal study showing that group sports participation predicted growth in inhibition, shift, and working memory from third to fourth grade.^36^ Our study adds value by examining more EF subscales, comparing team and individual sports, and including children who participated in both types of sports. Additionally, our observations align with a smaller study indicating that team sports athletes had better EF than self-paced sports athletes.^17^ The current study expanded results to a broader range of sports and a larger population. The consistent associations of team sports with EF may result from the sports’ inherent characteristics. Team sports are likely to incorporate high cognitive demands due to uncertainty from interactions with teammates and opponents; thus, players must rapidly and dynamically respond,^16^ such as by using inhibition to resist distraction from other players and working memory to recall locations.^37^ Consequently, team sports may serve as practice grounds for enhancing EF.

Soccer demonstrated consistent associations with better EF, aligning with previous studies that soccer interventions can enhance cognitive skills.^38,39^ Our study extended those findings by revealing associations between soccer and more aspects of EF. However, we observed no associations with inhibition. One reason may be the use of a questionnaire instead of a performance-based test in measurement. Another reason might be our study design: we compared EF in children playing soccer with that of children in other sports (eg, volleyball), whereas previous studies compared soccer interventions with nonintervention controls. The observations with regard to soccer and inhibition may be mitigated by the significant association between volleyball and inhibition.

Individual sports exhibited fewer positive associations with EF than did team sports, possibly because popular individual sports, like swimming and dance, are closed-skill sports.^36^ These activities involve repetitive and predictable contexts, which requires lower cognitive demands, thus affecting EF less.^40^ In this study, the association between swimming and behavioral regulation may have been influenced by the Dutch swimming education system. Most Dutch children start training at 4 to 5 years of age and complete it within 1 to 2 years, but those with behavioral challenges often start later and take longer, possibly remaining in training at ages 10 to 11 years. This may explain the negative association with EF at that age. Conversely, gymnastics, another closed-skill sport, was associated with better EF. Although a past study showed positive effects of gymnastics on EF,^41^ no studies to our knowledge have explored subscales, like organization and monitoring. Future research on EF subdomains is needed.

The negative association between martial arts and inhibition cannot be attributed to closed skills, as martial arts are open-skill sports typically associated with better EF.^42^ Disciplines like taekwondo and judo promote self-control,^43,44^ which may lead parents of children with inhibition issues to enroll them for improvement, potentially explaining the negative association observed in this study. Additionally, since the number of children participating in martial arts was small, conclusions about martial arts should be interpreted with caution.

Our study observed positive associations between total sports time and EF, which was likely due to repeated practice and constant cognitive challenges.^42^ More sports time translates to greater involvement and more opportunities to practice cognitive skills.^6^ Since total sports time included various sports with and without associations with EF, the associations of sports time with EF were significant but effect estimates were small.

### Limitations

This study has several limitations. Daily physical activity, sports participation, and EF were assessed at a single time point, which limited tracking changes over time and weakened the conclusions. Parental reporting of sports participation at 1 time point may have also introduced recall bias. Thus, future studies with multiple time points and more accurate measurements are warranted. Additionally, the small number of participants in certain sports, such as martial arts, makes conclusions uncertain. These associations should be investigated in larger samples.

## Conclusions

In this cohort study of Dutch children, early-life MVPA was not associated with EF in middle childhood. At ages 10 to 11 years, more sports time was associated with better EF. While associations varied among specific sports, children who participated in team sports consistently showed superior EF compared with those exclusively engaging in individual sports. These findings underscore the need to explore the effects of varying physical activity intensities on EF beyond MVPA and highlight that both quantity and type of sports may be crucial factors associated with EF.

## Notes

Supplement 1. **eTable 1.** Abbreviations and Definition of BRIEF Subscales **eFigure.** Study Design **eTable 2.** Cronbach α of BRIEF **eTable 3.** All Types of Sports **eTable 4.** Correlations Between Daily Physical Activity, Sports, and Executive Function **eTable 5.** BRIEF T Scores by Number and Type of Sports **eTable 6.** Association Between Daily Physical Activity and EF, Unadjusted **eTable 7.** Association Between Sport Types and EF, Unadjusted **eTable 8.** Sensitivity Analysis of Association Between Types of Sports and EF **eTable 9.** Association Between Types of Sports and EF, With Team Sports as Reference **eTable 10.** Association Between Types of Sports and EF, With No Sport as Reference

Supplement 2.
Data Sharing Statement

## References

[1] Chaput JP, Willumsen J, Bull F, . 2020 WHO guidelines on physical activity and sedentary behaviour for children and adolescents aged 5-17 years: summary of the evidence. Int J Behav Nutr Phys Act. 2020;17(1):141. doi:10.1186/s12966-020-01037-z 33239009 PMC7691077

[2] Erickson KI, Hillman C, Stillman CM, ; for 2018 Physical Activity Guidelines Advisory Committee. Physical activity, cognition, and brain outcomes: a review of the 2018 Physical Activity Guidelines. Med Sci Sports Exerc. 2019;51(6):1242-1251. doi:10.1249/MSS.0000000000001936 31095081 PMC6527141

[3] Diamond A. Executive functions. Annu Rev Psychol. 2013;64(1):135-168. doi:10.1146/annurev-psych-113011-143750 23020641 PMC4084861

[4] de Greeff JW, Bosker RJ, Oosterlaan J, Visscher C, Hartman E. Effects of physical activity on executive functions, attention and academic performance in preadolescent children: a meta-analysis. J Sci Med Sport. 2018;21(5):501-507. doi:10.1016/j.jsams.2017.09.595 29054748

[5] Liu S, Yu Q, Li Z, . Effects of acute and chronic exercises on executive function in children and adolescents: a systemic review and meta-analysis. Front Psychol. 2020;11:554915. doi:10.3389/fpsyg.2020.554915 33391074 PMC7773601

[6] Xue Y, Yang Y, Huang T. Effects of chronic exercise interventions on executive function among children and adolescents: a systematic review with meta-analysis. Br J Sports Med. 2019;53(22):1397-1404. doi:10.1136/bjsports-2018-099825 30737201

[7] Luo X, Herold F, Ludyga S, . Association of physical activity and fitness with executive function among preschoolers. Int J Clin Health Psychol. 2023;23(4):100400. doi:10.1016/j.ijchp.2023.100400 37663042 PMC10469079

[8] Zeng X, Cai L, Wong SH, . Association of sedentary time and physical activity with executive function among children. Acad Pediatr. 2021;21(1):63-69. doi:10.1016/j.acap.2020.02.027 32112865

[9] Wickel EE. Sedentary time, physical activity, and executive function in a longitudinal study of youth. J Phys Act Health. 2017;14(3):222-228. doi:10.1123/jpah.2016-0200 27918695

[10] Galle SA, Liu J, Bonnechère B, . The long-term relation between physical activity and executive function in the Rotterdam Study. Eur J Epidemiol. 2023;38(1):71-81. doi:10.1007/s10654-022-00902-4 36166135

[11] Sprengeler O, Buck C, Hebestreit A, Wirsik N, Ahrens W. Sports contribute to total moderate to vigorous physical activity in school children. Med Sci Sports Exerc. 2019;51(8):1653-1661. doi:10.1249/MSS.0000000000001948 30829902 PMC6693922

[12] Spanou M, Kaioglou V, Pesce C, Mavilidi MF, Venetsanou F. “Move” their brain: motor competence mediates the relationship of physical activity and executive functions in children. Appl Sci (Basel). 2022;12(20):10527. doi:10.3390/app122010527

[13] Kokko S, Martin L, Geidne S, . Does sports club participation contribute to physical activity among children and adolescents? a comparison across six European countries. Scand J Public Health. 2019;47(8):851-858. doi:10.1177/1403494818786110 29999480

[14] Overview of core indicators for sports and exercise. National Institute for Public Health and the Environment. Accessed July 3, 2024. https://www.sportenbewegenincijfers.nl/kernindicatoren

[15] Wang CH, Chang CC, Liang YM, . Open vs closed skill sports and the modulation of inhibitory control. PLoS One. 2013;8(2):e55773. doi:10.1371/journal.pone.0055773 23418458 PMC3572130

[16] Contreras-Osorio F, Campos-Jara C, Martínez-Salazar C, Chirosa-Ríos L, Martínez-García D. Effects of sport-based interventions on children’s executive function: a systematic review and meta-analysis. Brain Sci. 2021;11(6):755. doi:10.3390/brainsci11060755 34200362 PMC8226694

[17] De Waelle S, Laureys F, Lenoir M, Bennett SJ, Deconinck FJA. Children involved in team sports show superior executive function compared to their peers involved in self-paced sports. Children (Basel). 2021;8(4):264. doi:10.3390/children8040264 33808250 PMC8065925

[18] L’Abée C, Sauer PJ, Damen M, Rake JP, Cats H, Stolk RP. Cohort profile: the GECKO Drenthe study, overweight programming during early childhood. Int J Epidemiol. 2008;37(3):486-489. doi:10.1093/ije/dym218 18238823

[19] World Medical Association. World Medical Association Declaration of Helsinki: ethical principles for medical research involving human subjects. JAMA. 2013;310(20):2191-2194. doi:10.1001/jama.2013.281053 24141714

[20] Butte NF, Wong WW, Lee JS, Adolph AL, Puyau MR, Zakeri IF. Prediction of energy expenditure and physical activity in preschoolers. Med Sci Sports Exerc. 2014;46(6):1216-1226. doi:10.1249/MSS.0000000000000209 24195866 PMC4010568

[21] Wendel-Vos GCW, Schuit AJ, Saris WHM, Kromhout D. Reproducibility and relative validity of the short questionnaire to assess health-enhancing physical activity. J Clin Epidemiol. 2003;56(12):1163-1169. doi:10.1016/S0895-4356(03)00220-8 14680666

[22] Campbell N, Gaston A, Gray C, Rush E, Maddison R, Prapavessis H. The Short Questionnaire to Assess Health-Enhancing (SQUASH) Physical Activity in adolescents: a validation using doubly labeled water. J Phys Act Health. 2016;13(2):154-158. doi:10.1123/jpah.2015-0031 26104341

[23] Kunitoki K, Hughes D, Elyounssi S, . Youth team sports participation associates with reduced dimensional psychopathology through interaction with biological risk factors. Biol Psychiatry Glob Open Sci. 2023;3(4):875-883. doi:10.1016/j.bpsgos.2023.02.001 37881582 PMC10593891

[24] Smidts DP, Huizinga M. BRIEF executive function questionnaire: manual. 2010. Vrije Universiteit Amsterdam. Accessed February 7, 2024. https://research.vu.nl/en/publications/brief-executieve-functies-gedragsvragenlijst-handleiding

[25] Sijtsma A, Bocca G, L’abée C, Liem ET, Sauer PJJ, Corpeleijn E. Waist-to-height ratio, waist circumference and BMI as indicators of percentage fat mass and cardiometabolic risk factors in children aged 3-7 years. Clin Nutr. 2014;33(2):311-315. doi:10.1016/j.clnu.2013.05.010 23768783

[26] Zeng X, Cai L, Yang W, Tan W, Huang W, Chen Y. Association between the 24-hour movement guidelines and executive function among Chinese children. BMC Public Health. 2022;22(1):1017. doi:10.1186/s12889-022-13420-5 35596171 PMC9121539

[27] Schmidt M, Jäger K, Egger F, Roebers CM, Conzelmann A. Cognitively engaging chronic physical activity, but not aerobic exercise, affects executive functions in primary school children: a group-randomized controlled trial. J Sport Exerc Psychol. 2015;37(6):575-591. doi:10.1123/jsep.2015-0069 26866766

[28] Taylor RW, Williams SM, Farmer VL, Taylor BJ. Changes in physical activity over time in young children: a longitudinal study using accelerometers. PLoS One. 2013;8(11):e81567. doi:10.1371/journal.pone.0081567 24282607 PMC3839894

[29] Downing KL, Hinkley T, Timperio A, . Volume and accumulation patterns of physical activity and sedentary time: longitudinal changes and tracking from early to late childhood. Int J Behav Nutr Phys Act. 2021;18(1):39. doi:10.1186/s12966-021-01105-y 33731102 PMC7971959

[30] Yang L, Corpeleijn E, Hartman E. A prospective analysis of physical activity and mental health in children: the GECKO Drenthe cohort. Int J Behav Nutr Phys Act. 2023;20(1):114. doi:10.1186/s12966-023-01506-1 37749578 PMC10521540

[31] De Crescenzo F, Licchelli S, Ciabattini M, . The use of actigraphy in the monitoring of sleep and activity in ADHD: a meta-analysis. Sleep Med Rev. 2016;26:9-20. doi:10.1016/j.smrv.2015.04.002 26163053

[32] Willcutt EG, Doyle AE, Nigg JT, Faraone SV, Pennington BF. Validity of the executive function theory of attention-deficit/hyperactivity disorder: a meta-analytic review. Biol Psychiatry. 2005;57(11):1336-1346. doi:10.1016/j.biopsych.2005.02.006 15950006

[33] Mora-Gonzalez J, Esteban-Cornejo I, Cadenas-Sanchez C, . Physical fitness, physical activity, and the executive function in children with overweight and obesity. J Pediatr. 2019;208:50-56.e1. doi:10.1016/j.jpeds.2018.12.028 30902422

[34] Syväoja HJ, Tammelin TH, Ahonen T, Kankaanpää A, Kantomaa MT. The associations of objectively measured physical activity and sedentary time with cognitive functions in school-aged children. PLoS One. 2014;9(7):e103559. doi:10.1371/journal.pone.0103559 25061820 PMC4111611

[35] Horowitz-Kraus T, Hutton JS. Brain connectivity in children is increased by the time they spend reading books and decreased by the length of exposure to screen-based media. Acta Paediatr. 2018;107(4):685-693. doi:10.1111/apa.14176 29215151

[36] Koepp AE, Gershoff ET. Amount and type of physical activity as predictors of growth in executive functions, attentional control, and social self-control across 4 years of elementary school. Dev Sci. 2022;25(1):e13147. doi:10.1111/desc.13147 34240519 PMC8639632

[37] Howard SJ, Vella SA, Cliff DP. Children’s sports participation and self-regulation: bi-directional longitudinal associations. Early Child Res Q. 2018;42:140-147. doi:10.1016/j.ecresq.2017.09.006

[38] Huijgen BCH, Leemhuis S, Kok NM, . Cognitive functions in elite and sub-elite youth soccer players aged 13 to 17 years. PLoS One. 2015;10(12):e0144580. doi:10.1371/journal.pone.0144580 26657073 PMC4691195

[39] Dong X, Gui X, Klich S, . The effects of football juggling learning on executive function and brain functional connectivity. Front Hum Neurosci. 2024;18:1362418. doi:10.3389/fnhum.2024.1362418 38516307 PMC10954781

[40] Möhring W, Klupp S, Ludyga S, Grob A. Executive functions in children engaging in open- and closed-skilled sports. Psychol Sport Exerc. 2022;61:102218. doi:10.1016/j.psychsport.2022.102218

[41] Hsieh SS, Lin CC, Chang YK, Huang CJ, Hung TM. Effects of childhood gymnastics program on spatial working memory. Med Sci Sports Exerc. 2017;49(12):2537-2547. doi:10.1249/MSS.0000000000001399 28796655

[42] Diamond A, Lee K. Interventions shown to aid executive function development in children 4 to 12 years old. Science. 2011;333(6045):959-964. doi:10.1126/science.1204529 21852486 PMC3159917

[43] Lakes KD, Bryars T, Sirisinahal S, . The Healthy for Life Taekwondo Pilot Study: a preliminary evaluation of effects on executive function and BMI, feasibility, and acceptability. Ment Health Phys Act. 2013;6(3):181-188. doi:10.1016/j.mhpa.2013.07.002 24563664 PMC3927879

[44] Ludyga S, Mücke M, Leuenberger R, . Behavioral and neurocognitive effects of judo training on working memory capacity in children with ADHD: a randomized controlled trial. Neuroimage Clin. 2022;36:103156. doi:10.1016/j.nicl.2022.103156 35988343 PMC9402389

