# Supplementary material for: Daily Physical Activity, Sports Participation, and Executive Function in Children
Source: JAMA Netw Open. 2024 Dec 17;7(12):e2449879. doi: 10.1001/jamanetworkopen.2024.49879 (PMC11653117; doi:10.1001/jamanetworkopen.2024.49879)
Supplement: Supplement 1. — eTable 1. Abbreviations and Definition of BRIEF Subscales eFigure. Study Design eTable 2. Cronbach α of BRIEF eTable 3. All Types of Sports eTable 4. Correlations Between Daily Physical Activity, Sports, and Executive Function eTable 5. BRIEF T Scores by Number and Type of Sports eTable 6. Association Between Daily Physical Activity and EF, Unadjusted eTable 7. Association Between Sport Types and EF, Unadjusted eTable 8. Sensitivity Analysis of Association Between Types of Sports and EF eTable 9. Association Between Types of Sports and EF, With Team Sports as Reference eTable 10. Association Between Types of Sports and EF, With No Sport as Reference [file jamanetwopen-e2449879-s001.pdf]

Supplementary Online Content

Yang L, Corpeleijn E, Hartman E. Daily physical activity, sports participation, and executive function in children. *JAMA Netw Open*. 2024;7(12):e2449879. doi:10.1001/jamanetworkopen.2024.49879

eTable 1. Abbreviations and Definition of BRIEF Subscales
eFigure 1. Study Design
eTable 2. Cronbach α of BRIEF
eTable 3. All Types of Sports
eTable 4. Correlations Between Daily Physical Activity, Sports, and Executive Function
eTable 5. BRIEF T Scores by Number and Type of Sports
eTable 6. Association Between Daily Physical Activity and EF, Unadjusted
eTable 7. Association Between Sport Types and EF, Unadjusted
eTable 8. Sensitivity Analysis of Association Between Types of Sports and EF
eTable 9. Association Between Types of Sports and EF, With Team Sports as Reference
eTable 10. Association Between Types of Sports and EF, With No Sport as Reference

This supplementary material has been provided by the authors to give readers additional information about their work.

© 2024 Yang L et al. *JAMA Network Open*.

eTable 1. Abbreviations and Definition of BRIEF subscales

**Abbreviations**	
PA	Physical activity
SED	Sedentary behavior
LPA	Light physical activity
MPA	Moderate physical activity
VPA	Vigorous physical activity
MVPA	Moderate-to-vigorous physical activity
**BRIEF variables**	
Global Executive Composite, GEC	Overall summary score, reflects overall executive function
Behavioral Regulation Index, BRI	It assesses the ability control impulses and regulate emotional responses
Metacognition Index, MI	It assesses the ability to manage tasks by initiating, planning, organizing, and monitoring goal-oriented behaviors
Inhibition	Ability to control impulses and resist distractions
Shift	Capacity to adapt to changes and switch between tasks or thoughts
Emotional control	Ability to regulate emotional responses and manage frustration
Initiate	Ability to start tasks independently without prompting
Working memory	Ability to hold and manipulate information in mind for short periods
Plan/organize	Ability to develop strategies, prioritize tasks, and organize activities effectively
Organization of material	Ability to keep belongings and materials organized and orderly
Monitor	Tracking one’s own performance and self-evaluating to ensure tasks are completed correctly

© 2024 Yang L et al. *JAMA Network Open*.

eFigure 1. Study design

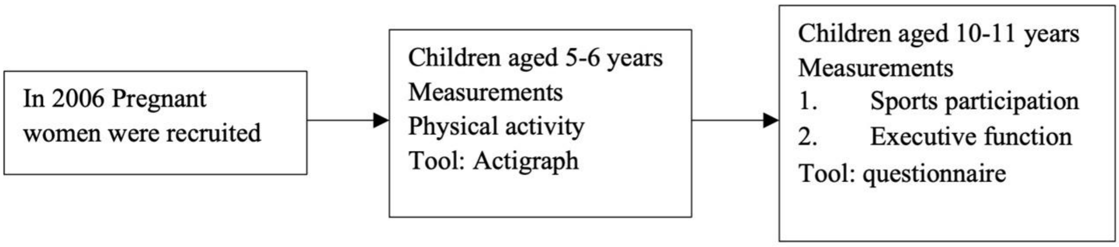

© 2024 Yang L et al. *JAMA Network Open*.

eTable 2. Cronbach α of BRIEF

BRIEF	Cronbach's Alpha
**Index**	
Global Executive Composite	0.97
Behavioral Regulation Index	0.93
Metacognition Index	0.96
**Subscales**	
Inhibition	0.86
Shift	0.83
Emotional control	0.89
Initiate	0.83
Working memory	0.91
Plan/organize	0.87
Organization of materials	0.86
Monitor	0.85

© 2024 Yang L et al. *JAMA Network Open*.

eTable 3. All types of sports

**Team sports**	basketball, football, frisbee, handball, hockey, korfball, majorette, rugby, scouting, volleyball, water polo
**Individual sports**	athletics, badminton, BMX/MTB, bowling, cycling, dancing, fitness, golf, gymnastics, horse riding, trampoline jump, martial arts, motocross, roller skating, running, sailing, skating, survival, swimming, table tennis, tennis, and walking

© 2024 Yang L et al. *JAMA Network Open*.

eTable 4. Correlations between daily physical activity, sports, and BRIEF T scores

PA and sports	GEC	BRI	MI	Inhibition	Shift	Emotional control	Initiate	Working memory	Plan/ organize	Organization of material	Monitor
**PA at ages 5-6**											
SED [a]	-0.057	-0.093	-0.038	-0.109	-0.033	-0.076	0.003	-0.047	-0.027	-0.036	-0.094
P	.19	.03	.38	.01	.46	.08	.94	.28	.54	.41	.03
LPA a	0.031	0.040	0.026	.086	-0.033	0.018	-0.014	0.031	0.012	0.040	.086
P	.49	.36	.56	.05	.45	.68	.75	.47	.78	.36	.05
MPA [a]	0.047	0.060	0.034	0.067	0.000	0.064	-0.044	0.025	0.026	0.047	.095
P	.28	.17	.43	.12	>.99	.14	.31	.57	.56	.28	.03
VPA [a]	-0.036	-0.026	-0.034	-0.013	-0.073	-0.008	-0.070	-0.013	-0.036	-0.015	-0.005
P	.42	.55	.44	.77	.10	.86	.11	.76	.41	.73	.91
MVPA [a]	0.014	0.025	0.007	0.038	-0.031	0.033	-0.056	0.011	-0.001	0.024	0.053
P value	.76	.57	.87	.39	.48	0.45	.20	.80	>. 99	.58	.23
**Sports at ages 10-11**											
Sport time [b]	-0.063	-0.070	-0.059	-0.041	-0.086	-0.073	-0.064	-0.075	-0.044	-0.026	-0.032
P	.07	.04	.09	.24	.01	.04	.07	.03	.21	.46	.36
Number of sports [b]	-0.017	-0.026	-0.012	-0.018	-0.028	-0.029	-0.008	-0.006	-0.002	-0.041	-0.010
P	.62	.45	.72	.60	.43	.40	.82	.87	.95	.24	.78
Type of sports [b]	-0.101	-0.123	-0.084	-0.095	-0.109	-0.117	-0.089	-0.074	-0.050	-0.084	-0.067
P	.004	<0.001	0.02	.006	.002	<.001	.01	.03	.15	.02	.05
**Specific sports at ages 10-11**											
Football	-0.118	-0.102	-0.117	-0.039	-0.097	-0.081	-0.103	-0.147	-0.080	-0.091	-0.082

© 2024 Yang L et al. *JAMA Network Open*.

PA and sports	GEC	BRI	MI	Inhibition	Shift	Emotional control	Initiate	Working memory	Plan/ organize	Organization of material	Monitor
P	.001	.003	.001	.27	.005	.02	.003	<.001	.02	.009	.02
Gymnastics	-0.071	-0.049	-0.077	-0.053	-0.012	-0.072	-0.021	-0.052	-0.065	-0.080	-0.091
P	.04	.16	.03	.13	.72	.04	.55	.14	.06	.02	.01
Dancing	-0.003	0.003	-0.004	0.016	-0.023	-0.017	-0.011	0.029	0.010	0.008	-0.036
P	.94	.94	.91	.65	.51	.62	.76	.40	.77	.82	.30
Martial arts	0.075	0.072	0.072	0.074	0.055	0.066	0.052	.072	0.065	0.041	0.047
P	.03	.04	.04	.03	.11	.06	.14	.04	.06	.24	.18
Horse-riding	0.044	0.015	0.058	0.030	0.009	-0.013	0.050	0.061	0.055	0.003	.080
P	.21	.66	.09	.39	.81	.71	.15	.08	.11	.94	.02
Swimming	0.057	.069	0.045	0.067	0.045	0.061	0.051	0.033	0.023	0.017	0.062
P	.10	.05	.20	.06	.20	.08	.14	.34	.50	.63	.08
Volleyball	-0.053	-0.071	-0.040	-0.091	-0.050	-0.059	-0.003	0.016	-0.044	-0.061	-0.081
P	.13	.04	.25	.01	.15	.09	.92	.65	.21	.08	.02
Tennis	-0.011	-0.023	-0.001	-0.070	0.005	0.002	0.018	-0.018	-0.002	0.023	-0.029
P	.75	.51	.98	.05	.88	.96	.60	.60	.10	.52	.40
Hockey	-0.043	-0.059	-0.033	-0.070	-0.035	-0.057	-0.056	-0.007	-0.026	0.012	-0.037
P	.22	.09	.35	.05	.31	.10	.11	.84	.46	.73	.29

Abbreviation: GEC=Global Executive Composite; BRI=behavioral regulation index; MI=metacognition index.

[a] SED: sedentary behaviors; LPA: light physical activity; MPA: moderate physical activity; VPA: vigorous physical activity; MVPA: moderate-to-vigorous physical activity.

[b] time spent in sports (h/w).

[c] the number of sports children engaged in with range 0 to 4.

[d] the type of sports: 0=no sport, 1=individual sports, 2=team sports, 3=both sports.

© 2024 Yang L et al. *JAMA Network Open*.

eTable 5. BRIEF T scores by number of sports and type of sports

BRIEF			Number of Sports [a]			
**BRIEF, mean (SD)**	**0**	**1**	**2**	**3**	**4**	***P value*** [c]
GEC	40.88 (10.58)	41.25 (10.06)	40.21 (9.25)	40.38 (8.75)	41.31 (8.25)	0.74
BRI	43.92 (10.46)	43.72 (9.33)	42.24 (8.76)	43.87 (8.65)	43.44 (7.88)	0.34
MI	40.62 (9.87)	41.25 (10.09)	40.54 (9.21)	40.02 (8.66)	41.69 (8.44)	0.79
			**Type of sports** [b]			
**BRIEF, mean (SD)**	**No sport**	**Individual**	**Team**	**Both**		*P value* [c]
GEC	40.88 (10.58)	42.62 (9.94)	39.59 (9.44)	39.72 (9.02)		0.001
BRI	43.92 (10.46)	45.18 (9.44)	41.82 (8.57)	42.28 (8.64)		< 0.001
MI	40.62 (9.87)	42.42 (9.91)	39.91 (9.68)	39.86 (8.84)		0.005

Abbreviation: GEC=Global Executive Composite; BRI=behavioral regulation index; MI=metacognition index.

[a] the number of sports: children were grouped by the number of sports children engaged in with range 0 to 4; [b] type of sports: children were grouped by the type of sports they engaged in, including no sport, individual sports, team sports, and both sports; [c] ANOVA was used to compare across groups.

© 2024 Yang L et al. *JAMA Network Open*.

eTable 6. Associations between daily physical activity and EF (unadjusted)

BRIEF	SED		LPA		MVPA		MPA		VPA	
	B	95% CI	B	95% CI	B	95% CI	B	95% CI	B	95% CI
**Index** [a]										
GEC	-0.29	(-0.74, 0.17)	0.28	(-0.40, 0.96)	0.00	(-0.35, 0.34)	0.28	(-0.28, 0.84)	-0.42	(-1.10, 0.26)
BRI	0.22	(-0.78, 0.08)	0.33	(-0.29, 1.00)	0.17	(-0.34, 0.31)	0.27	(-0.23, 0.84)	0.33	(-1.15, 0.15)
MI	-0.25	(-0.71, 0.21)	0.27	(-0.40, 0.95)	0.02	(-0.32, 0.36)	0.25	(-0.31, 0.81)	-0.30	(-0.98, 0.38)
**Subscales** [a]										
Inhibition	-0.53	(-0.93, -0.12)	0.73	(0.13, 1.32)	0.11	(-0.20, 0.41)	0.44	(-0.06, 0.93)	-0.23	(-0.83, 0.38)
Shift	0.00	(-0.43, 0.43)	-0.23	(-0.87, 0.41)	-0.20	(-0.52, 0.13)	-0.05	(-0.58, 0.48)	-0.70	(-1.34, -0.06)
Emotional control	-0.30	(-0.71, 0.11)	0.27	(-0.35, 0.88)	0.09	(-0.22, 0.40)	0.42	(-0.09, 0.93)	-0.27	(-0.89, 0.35)
Initiate	0.00	(-0.46, 0.47)	-0.04	(-0.73, 0.65)	-0.18	(-0.53, 0.17)	-0.27	(-0.84, 0.31)	-0.33	(-1.02, 0.37)
Working memory	-0.25	(-0.69, 0.19)	0.23	(-0.43, 0.88)	0.01	(-0.32, 0.35)	0.19	(-0.35, 0.74)	-0.23	(-0.89, 0.43)
Plan/organize	-0.22	(-0.68, 0.24)	0.19	(-0.49, 0.87)	0.07	(-0.28, 0.41)	0.33	(-0.24, 0.89)	-0.22	(-0.90, 0.47)
Organization of material	-0.14	(-0.57, 0.29)	0.28	(-0.35, 0.92)	0.03	(-0.29, 0.35)	0.21	(-0.32, 0.73)	-0.18	(-0.82, 0.46)
Monitor	-0.45	(-0.91, 0.00)	0.64	(-0.03, 1.31)	0.13	(-0.21, 0.47)	0.52	(-0.04, 1.07)	-0.25	(-0.93, 0.43)

Abbreviation: SED: sedentary behaviors; LPA: light physical activity; MPA: moderate physical activity; VPA: vigorous physical activity; MVPA: moderate-to-vigorous physical activity; GEC=Global Executive Composite; BRI=behavioral regulation index; MI=metacognition index.

[a] Unadjusted models

© 2024 Yang L et al. *JAMA Network Open*.

eTable 7. Association between sport types and EF (unadjusted)

BRIEF	No Sport vs Individual		Team Sport vs Individual		Both vs Individual	
	MD (SE)	*p*	MD (SE)	*P*	MD (SE)	*p*
**Index** [a, b]						
Overall	-1.73 (1.44)	0.23	-2.99 (0.80)	<0.001	-2.89 (0.89)	0.001
BRI	-1.26 (1.36)	0.36	-3.35 (0.75)	<0.001	-2.90 (0.84)	0.001
MI	-1.81 (1.44)	0.21	-2.48 (0.80)	0.002	-2.55 (0.89)	0.004
**Subscales** [a, b]						
Inhibition	-0.49 (1.25)	0.69	-2.11 (0.70)	0.002	-1.99 (0.77)	0.01
Flexibility	-0.79 (1.35)	0.56	-2.78 (0.75)	<0.001	-2.41 (0.83)	0.004
Emotional control	-1.28 (1.26)	0.31	-2.63 (0.70)	<0.001	-2.35 (0.78)	0.003
Initiate	-1.68 (1.46)	0.25	-2.75 (0.81)	0.001	-2.48 (0.90)	0.006
Working memory	-2.03 (1.36)	0.14	-2.15 (0.76)	0.004	-2.16 (0.84)	0.01
Plan/organize	-2.01 (1.43)	0.16	-1.77 (0.80)	0.03	-1.63 (0.88)	0.07
Organization of material	-0.36 (1.29)	0.78	-1.40 (0.72)	0.05	-1.91 (0.79)	0.02
Monitor	-1.47 (1.43)	0.30	-2.23 (0.79)	0.005	-1.90 (0.88)	0.03

Abbreviation: GEC=Global Executive Composite; BRI=behavioral regulation index; MI=metacognition index.

[a] Take ‘individual sport’ as reference group, number of children is 823. [b] Unadjusted models.

© 2024 Yang L et al. *JAMA Network Open*.

eTable 8. Sensitivity analysis: Association between type of sports and EF

BRIEF		No Sport vs Individual		Team Sport vs Individual		Both vs Individual	
		**MD (SE)**	***P***	**MD (SE)**	***P***	**MD (SE)**	***P***
**Index**							
GEC	Unadjusted	-1.40 (1.51)	0.36	-2.63 (0.92)	0.004	-3.32 (1.13)	0.003
	Adjusted	-1.29 (1.57)	0.41	-2.73 (0.94)	0.004	-3.21 (1.19)	0.007
BRI	Unadjusted	-1.14 (1.41)	0.42	-3.23 (0.86)	<0.001	-3.38 (1.06)	0.002
	Adjusted	-1.20 (1.48)	0.42	-3.29 (0.89)	<0.001	-3.30 (1.12)	0.003
MI	Unadjusted	-1.39 (1.51)	0.36	-2.02 (0.92)	0.028	-2.95 (1.13)	0.009
	Adjusted	-1.26 (1.56)	0.42	-2.18 (0.94)	0.021	-2.84 (1.18)	0.02
**Subscales**							
Inhibition	Unadjusted	-0.12 (1.32)	0.93	-1.76 (0.80)	0.029	-2.00 (0.99)	0.043
	Adjusted	-0.50 (1.37)	0.72	-1.98 (0.83)	0.017	-1.93 (1.04)	0.06
Shift	Unadjusted	-0.70 (1.41)	0.62	-2.66 (0.86)	0.002	-2.80 (1.06)	0.009
	Adjusted	-0.53 (1.46)	0.72	-2.49 (0.88)	0.005	-2.56 (1.11)	0.02
Emotional control	Unadjusted	-1.35 (1.32)	0.31	-2.70 (0.80)	0.001	-3.01 (0.99)	0.002
	Adjusted	-1.32 (1.38)	0.34	-2.80 (0.83)	0.001	-3.08 (1.04)	0.003
Initiate	Unadjusted	-1.50 (1.51)	0.32	-2.53 (0.92)	0.006	-3.61 (1.13)	0.002
	Adjusted	-0.69 (1.51)	0.65	-2.67 (0.91)	0.003	-3.53 (1.14)	0.002
Working memory	Unadjusted	-1.52 (1.42)	0.28	-1.62 (0.87)	0.062	-2.10 (1.06)	0.05
	Adjusted	-1.86 (1.48)	0.21	-1.56 (0.89)	0.079	-1.68 (1.12)	0.13
Plan/organize	Unadjusted	-1.84 (1.51)	0.23	-1.58 (0.92)	0.089	-2.30 (1.14)	0.04
	Adjusted	-1.45 (1.56)	0.35	-1.80 (0.94)	0.056	-2.48 (1.18)	0.04
Organization of material	Unadjusted	-0.46 (1.38)	0.74	-1.48 (0.84)	0.080	-2.24 (1.03)	0.03
	Adjusted	-0.11 (1.44)	0.94	-1.81 (0.86)	0.037	-2.61 (1.09)	0.02

© 2024 Yang L et al. *JAMA Network Open*.

BRIEF		No Sport vs Individual		Team Sport vs Individual		Both vs Individual	
		MD (SE)	***P***	MD (SE)	***P***	MD (SE)	***P***
Monitor	Unadjusted	-1.05 (1.48)	0.48	-1.77 (0.91)	0.05	-2.29 (1.11)	0.04
	Adjusted	-0.11 (1.44)	0.94	-1.81 (0.86)	0.04	-2.61 (1.09)	0.02

Abbreviation: MD (SE)= mean difference with standard error; GEC=Global Executive Composite; BRI=behavioral regulation index; MI=metacognition index.

[a] Take 'individual sport' as reference group.

[b] Adjusting for the exact age, BMI, outdoor play time, computer time, maternal education level, number of siblings, and time spent in sports.

[c] Number of children is 623, excluding children in swimming, martial arts, and horse riding due to their contracting associations with EF

© 2024 Yang L et al. *JAMA Network Open*.

eTable 9. Association between types of sports and EF (reference=team sports)

BRIEF		No vs Team		Individual vs Team		Both vs Team	
		MD (SE)	***P***	MD (SE)	***P***	MD (SE)	***P***
**Index [a]**							
Overall	Unadjusted	1.26 (1.44)	0.39	2.99 (0.80)	0.388	0.10 (0.91)	0.91
	Adjusted	1.48 (1.51)	0.33	3.03 (0.81)	<0.001	0.37 (0.94)	0.69
BRI	Unadjusted	2.09 (1.37)	0.13	3.35 (0.75)	<0.001	0.45 (0.86)	0.60
	Adjusted	2.20 (1.43)	0.12	3.39 (0.77)	<0.001	0.66 (0.89)	0.46
MI	Unadjusted	0.67 (1.45)	0.65	2.48 (0.80)	0.002	-0.08 (0.91)	0.93
	Adjusted	0.92 (1.50)	0.54	2.55 (0.81)	0.002	0.21 (0.93)	0.82
**Subscales [a]**							
Inhibition	Unadjusted	1.62 (1.27)	0.20	2.11 (0.70)	0.002	0.12 (0.80)	0.88
	Adjusted	1.78 (1.32)	0.18	2.29 (0.71)	0.001	0.32 (0.82)	0.69
Flexibility	Unadjusted	1.99 (1.36)	0.15	2.78 (0.75)	<0.001	0.37 (0.86)	0.67
	Adjusted	1.82 (1.42)	0.20	2.64 (0.76)	0.001	0.59 (0.88)	0.50
Emotional control	Unadjusted	1.35 (1.27)	0.29	2.63 (0.70)	<0.001	0.28 (0.80)	0.73
	Adjusted	1.55 (1.33)	0.24	2.72 (0.71)	<0.001	0.43 (0.82)	0.60
Initiate	Unadjusted	1.07 (1.47)	0.47	2.75 (0.81)	0.001	0.27 (0.92)	0.77
	Adjusted	1.66 (1.47)	0.26	2.73 (0.79)	0.001	0.55 (0.91)	0.54
Working memory	Unadjusted	0.12 (1.38)	0.93	2.15 (0.76)	0.004	-0.01 (0.86)	0.99
	Adjusted	-0.14 (1.43)	0.92	2.05 (0.77)	0.008	0.38 (0.89)	0.67
Plan/organize	Unadjusted	-0.24 (1.45)	0.87	1.77 (0.80)	0.027	0.14 (0.91)	0.88
	Adjusted	0.11 (1.49)	0.94	1.82 (0.80)	0.023	0.33 (0.93)	0.72

© 2024 Yang L et al. *JAMA Network Open*.

BRIEF		No vs Team		Individual vs Team		Both vs Team	
		MD (SE)	***P***	MD (SE)	***P***	MD (SE)	***P***
Organization of material	Unadjusted	1.04 (1.30)	0.42	1.40 (0.72)	0.05	-0.51 (0.82)	0.53
	Adjusted	1.56 (1.36)	0.25	1.71 (0.73)	0.02	-0.50 (0.84)	0.55
Monitor	Unadjusted	0.76 (1.45)	0.60	2.23 (0.79)	0.005	0.33 (0.91)	0.71
	Adjusted	1.02 (1.51)	0.50	2.25 (0.81)	0.005	0.48 (0.93)	0.61

Abbreviation: MD (SE)= mean difference with standard error; GEC=Global Executive Composite; BRI=behavioral regulation index; MI=metacognition index.

[a] Adjusting for the exact age, BMI, outdoor play time, computer time, maternal education level, number of siblings, and time spent in sports

© 2024 Yang L et al. *JAMA Network Open*.

eTable 10. Association between types of sports and EF (reference=no sport)

BRIEF		Individual vs No		Team vs No		Both vs No	
		MD (SE)	*P*	MD (SE)	*P*	MD (SE)	*P*
**Index [a]**							
GEC	Unadjusted	1.73 (1.44)	0.23	-1.26 (1.46)	0.40	-1.16 (1.51)	0.44
	Adjusted	1.55 (1.48)	0.30	-1.48 (1.51)	0.33	-1.11 (1.63)	0.50
BRI	Unadjusted	1.26 (1.36)	0.36	-2.09 (1.37)	0.13	-1.64 (1.42)	0.25
	Adjusted	1.19 (1.40)	0.40	-2.20 (1.43)	0.12	-1.54 (1.54)	0.32
MI	Unadjusted	1.81 (1.44)	0.21	-0.67 (1.45)	0.65	-0.74 (1.50)	0.62
	Adjusted	1.62 (1.48)	0.27	-0.92 (1.50)	0.54	-0.71 (1.63)	0.66
**Subscales [a]**							
Inhibition	Unadjusted	0.49 (1.25)	0.69	-1.62 (1.27)	0.20	-1.50 (1.31)	0.25
	Adjusted	0.51 (1.30)	0.70	-1.78 (1.32)	0.18	-1.45 (1.43)	0.31
Shift	Unadjusted	0.79 (1.35)	0.56	-1.99 (1.36)	0.15	-1.62 (1.41)	0.25
	Adjusted	0.81 (1.39)	0.56	-1.82 (1.42)	0.20	-1.23 (1.53)	0.42
Emotional control	Unadjusted	1.28 (1.26)	0.31	-1.35 (1.27)	0.29	-1.07 (1.32)	0.42
	Adjusted	1.16 (1.30)	0.37	-1.55 (1.33)	0.24	-1.12 (1.44)	0.44
Initiate	Unadjusted	1.68 (1.46)	0.25	-1.07 (1.47)	0.47	-0.80 (1.52)	0.60
	Adjusted	1.08 (1.44)	0.46	-1.66 (1.47)	0.26	-1.10 (1.59)	0.49
Working memory	Unadjusted	2.03 (1.36)	0.14	-0.12 (1.38)	0.93	-0.13 (1.42)	0.93
	Adjusted	2.19 (1.40)	0.12	0.14 (1.43)	0.92	0.52 (1.55)	0.73
Plan/organize	Unadjusted	2.01 (1.43)	0.16	0.24 (1.45)	0.87	0.37 (1.50)	0.80
	Adjusted	1.71 (1.46)	0.24	-0.11 (1.49)	0.94	0.22 (1.61)	0.89
Organization of material	Unadjusted	0.36 (1.29)	0.78	-1.04 (1.30)	0.42	-1.55 (1.35)	0.25
	Adjusted	0.15 (1.33)	0.91	-1.56 (1.36)	0.25	-2.07 (1.47)	0.16
Monitor	Unadjusted	1.47 (1.43)	0.30	-0.76 (1.45)	0.60	-0.43 (1.49)	0.78

© 2024 Yang L et al. *JAMA Network Open*.

BRIEF		Individual vs No		Team vs No		Both vs No	
		MD (SE)	***P***	MD (SE)	***P***	MD (SE)	***P***
	Adjusted	1.23 (1.48)	0.41	-1.02 (1.51)	0.50	-0.55 (1.63)	0.74

Abbreviation: MD (SE)= mean difference with standard error; GEC=Global Executive Composite; BRI=behavioral regulation index; MI=metacognition index.

[a] Adjusting for the exact age, BMI, outdoor play time, computer time, maternal education level, number of siblings, and time spent in sports

© 2024 Yang L et al. *JAMA Network Open*.
