# Supplementary material for: Daily Physical Activity, Sports Participation, and Executive Function in Children
Source: JAMA Netw Open. 2024 Dec 17;7(12):e2449879. doi: 10.1001/jamanetworkopen.2024.49879 (PMC11653117; doi:10.1001/jamanetworkopen.2024.49879)
Supplement: Supplement 2. — Data Sharing Statement [file jamanetwopen-e2449879-s002.pdf]

Data Sharing Statement

Yang. Daily Physical Activity, Sports Participation, and Executive Function in Children. *JAMA Netw Open*. Published December 17, 2024. doi:10.1001/jamanetworkopen.2024.49879

Data
Data available: No

Additional Information
Explanation for why data not available: Corpeleijn, E had full access to all the data in the study and takes responsibility for the integrity of the data and the accuracy of the data analysis. The datasets of the current study are not publicly available due to data restrictions indicated in the informed consent forms. However, they can be obtained from the author on reasonable request.
